# The association between social support and quality of life in patients with severe stroke: a chain mediation analysis of resilience and self-efficacy

**DOI:** 10.3389/fpsyt.2026.1851981

**Published:** 2026-07-16

**Authors:** Yun Li, Yuru Liu, Dan Liu, Hongmei Pang, Rongrong Hao, Juan Du, Xiaonan Liu, Yining Shi

**Affiliations:** 1 Department of Intensive Care Rehabilitation, Shandong Provincial Public Health Clinical Center, Jinan, Shandong, China; 2 Center of Critical Care Medicine, Shandong Provincial Public Health Clinical Center, Jinan, Shandong, China; 3 Offline Center, Shandong Provincial Public Health Clinical Center, Jinan, Shandong, China; 4 Surgical Intensive Care Unit, Shandong Provincial Public Health Clinical Center, Jinan, Shandong, China; 5 Department of Gastroenterology, Shandong Provincial Public Health Clinical Center, Jinan, Shandong, China

**Keywords:** quality of life, resilience, self-efficacy, social support, stroke

## Abstract

**Objective:**

To explore the chain mediating effects of resilience and self-efficacy on the association between social support and quality of life (QoL) among patients with severe stroke.

**Methods:**

This cross-sectional study enrolled 272 inpatients between June 2023 and June 2025. Validated instruments were used to assess social support (SSRS), resilience (CD-RISC), self-efficacy (GSES), and quality of life (QoL) (SS-QOL). PROCESS Model 6 with 5,000 bias-corrected bootstrap samples was used to test sequential mediation while adjusting for key covariates.

**Results:**

Mean scores indicated moderate levels of social support, resilience, self-efficacy, and QoL. Quality of life scores were significantly higher among patients aged <60 years compared to those aged ≥60 years (54.62 ± 14.07 vs. 49.34 ± 13.12), among patients with higher education levels (college or above: 54.73 ± 14.27 vs. primary school or below: 47.26 ± 11.38), among patients covered by Employee Health Insurance compared to self-funded patients (53.26 ± 13.42 vs. 42.36 ± 11.25), and among patients with lower NIHSS scores (10–14 points: 56.38 ± 13.45 vs. 15–24 points: 46.44 ± 12.41). All comparisons were statistically significant at *P* < 0.05. All main variables were positively correlated. The direct association between social support and QoL was significant (adjusted unstandardized β = 0.88, 95% CI [0.790, 0.975]), accounting for 53.5% of the total association. The three indirect associations were: (1) SSRS - CD-RISC - SS-QOL: β = 0.255, 95% CI [0.192, 0.329]; (2) SSRS - GSES - SS-QOL: β = 0.126, 95% CI [0.020, 0.208]; and (3) SSRS - CD-RISC - GSES - SS-QOL: β = 0.385, 95% CI [0.289, 0.510],supporting the hypothesized chain mediation model.

**Conclusion:**

Social support was strongly associated with QoL, both directly and indirectly through resilience and self-efficacy and their sequential pathways. These findings highlight potential intervention targets, although causal inferences are limited by the study’s cross-sectional design. Strengthening social support and related psychological resources may improve rehabilitation outcomes in patients with severe strokes.

## 1 Introduction

Stroke is a leading cause of disability and mortality among adults worldwide. Severe stroke is characterized by rapid onset, severe neurological impairment, multiple complications, and high rates of disability and mortality. Patients often experience various sequelae, including hemiplegia, speech disorders, and cognitive decline, which seriously impair physical functioning and reduce quality of life (QoL) (1, 2). Social support, defined as the material and emotional support obtained from external sources, is an important external resource for coping with disease-related stress and maintaining physical and mental health. Previous studies have shown that adequate social support is associated with better rehabilitation adherence, alleviate negative emotions, and enhance functional outcomes in patients with stroke (3, 4). However, as an external environmental factor, social support may be associated with health outcomes through patients’ internal psychological processes. The specific mechanisms underlying this association remain insufficiently understood.

Recent research in the Chinese context has increasingly recognized the importance of psychosocial factors in stroke rehabilitation. For instance, a multi-center study among Chinese stroke survivors found that health literacy, social support, and resilience collectively explained a substantial proportion of variance in QoL outcomes, with resilience emerging as a particularly strong mediator (8). Furthermore, research has demonstrated that self-efficacy plays a critical role in the rehabilitation process among Chinese patients with stroke, mediating the relationship between psychological distress and functional recovery (21). In the context of severe stroke specifically, Olsson et al. (17) found that both self-efficacy and resilience were significantly lower in patients with severe aphasia compared to those with milder impairments, suggesting that these psychological resources may be especially relevant for patients with greater neurological deficits.

Beyond the Chinese context, international research has identified social support as a consistent predictor of QoL in stroke populations. A recent systematic review by Gurková et al. (23) demonstrated that social support was independently associated with better functional outcomes and health-related QoL at three months post-stroke, even after adjusting for clinical severity. Similarly, Bellinger et al. (7) showed that depressive symptoms, self-efficacy, and physical activity were dynamically interrelated over time, highlighting the potential for interventions targeting these modifiable factors. Research from diverse healthcare settings has also documented that resilience serves as a buffer against the negative effects of disability on psychological well-being (22), while social support may enhance resilience and self-efficacy through mechanisms of verbal encouragement and observational learning (5).

Resilience refers to an individual’s capacity for psychological adaptation and recovery in the face of adversity, trauma, disease, and other stressful events. It is considered a key internal psychological resource linking external support to individual health behaviors (5). Self-efficacy refers to an individual’s belief in their ability to successfully perform a behavior and achieve a specific goal, which can directly influence rehabilitation behaviors, disease management, and health outcomes (6). Previous studies have demonstrated that resilience and self-efficacy independently serve as mediators in the relationship between social support and QoL in stroke (7, 8). However, limited research has explored the potential sequential mediating pathways between these two psychological factors.

Therefore, this study aimed to investigate the chain-mediation effects of resilience and self-efficacy on the relationship between social support and QoL among patients with severe stroke. By systematically analyzing the specific pathways and mechanisms through which social support influences QoL, this study aims to provide a theoretical basis for the development of targeted psychological interventions and comprehensive rehabilitation support systems in clinical practice.

## 2 Data and methods

### 2.1 Research object

This study was approved by the Medical Ethics Committee of our hospital and was conducted in accordance with the Declaration of Helsinki. Written informed consent was obtained from all participants or their legally authorized representatives prior to enrollment. A total of 272 patients with severe stroke who were admitted to our hospital between June 2023 and June 2025 were selected as study participants.

The inclusion criteria were as follows: (1) meeting the diagnostic criteria for severe stroke (9) and confirmed by CT or MRI; (2) National Institutes of Health Stroke Scale (NIHSS) (10) score ≥10; (3) age between 18 and 80 years; (4) being 1–6 months after stroke onset, with stable vital signs (body temperature, heart rate, respiration, and blood pressure), no progression of neurological deficits, no requirement for intensive care, emergency surgery, or invasive rescue treatment, and ability to cooperate with rehabilitation training and questionnaire investigation; (5) absence of severe cognitive, speech, hearing, or visual impairments. Patients with mild hemiplegia or writing difficulties were eligible if they could clearly express their intentions independently after the researcher read the questionnaire items without guidance, and the questionnaire could be completed truthfully; (6) provision of written informed consent by the patient or family members. For patients who were unable to provide written informed consent due to severe physical impairments (e.g., hemiplegia affecting writing ability) or cognitive limitations that compromised decisional capacity, informed consent was obtained from their legally authorized representatives (family members or legal guardians). All participants or their representatives were provided with comprehensive information about the study purpose, procedures, potential risks, and the voluntary nature of participation before providing written consent.

The exclusion criteria were as follows: (1) history of prior stroke (ischemic or hemorrhagic) with residual severe neurological dysfunction that could confound the current functional and QoL assessments; (2) end-stage malignant tumors, severe hematological diseases, or severe active autoimmune diseases with an expected survival time of less than 6 months; (3) uncontrolled severe infections, including severe pulmonary infection, sepsis, intracranial infection, or multidrug-resistant bacterial infection, resulting in unstable clinical conditions; (4) complications after stroke onset, such as gastrointestinal bleeding, symptomatic pulmonary embolism, cerebral hernia, hydrocephalus, severe pressure ulcers, or refractory status epilepticus, which prevented completion of the investigation; and (5) a history of schizophrenia, bipolar disorder, major depressive disorder, or other severe psychiatric disorders, family history of major psychiatric disorders, or long-term use of antipsychotic medications.

The main variables included in this study were four core variables: social support, resilience, self-efficacy, and QoL, along with demographic and disease-related control variables. Fewer than 10 independent variables were included in the regression model. According to the principle of requiring at least 15 participants per variable, the minimum required sample size was estimated to be 150 participants. Considering the complexity of the chain mediation model and the possibility of invalid questionnaires, a sample size of 200–300 participants was recommended for bootstrap mediation analysis with bias correction, assuming a medium effect size (*r* ≈ 0.26), statistical power of 0.80, and significance level of α = 0.05. Based on these considerations, together with the actual survey conditions and an estimated invalid questionnaire rate of approximately 10%, the final target sample size was determined to be 270 participants. A total of 272 participants were ultimately included, which met the statistical requirements for mediation effect analysis.

### 2.2 Data collection instruments

#### 2.2.1 Participant information sheet

The participant information sheet was designed by the researchers and included demographic, sociological, and disease-related information, such as sex, age, marital status, education level, medical payment method, stroke type (ischemic/hemorrhagic), disease duration, and NIHSS score. The medical payment method was categorized into three types: Employee Health Insurance (basic medical insurance for urban employees, with higher reimbursement rates), Resident Health Insurance (basic medical insurance for urban and rural non-employed residents, with moderate reimbursement rates), and self-funded (patients paying all medical costs out-of-pocket without insurance coverage).

#### 2.2.2 Social Support Rating Scale

The Social Support Rating Scale (SSRS), developed by Shuiyuan (11), is one of the most widely used social support assessment tools in China. It includes three dimensions: objective support (three items), subjective support (four items), and support utilization (three items), with a total of 10 items. Each item is scored on a 1–4 scale, with total scores ranging from 12–66. Higher scores indicate higher levels of social support. In this study, Cronbach’s α coefficient of the scale was 0.892.

#### 2.2.3 Connor–Davidson Resilience Scale

The revised Chinese version of the Connor–Davidson Resilience Scale (CD-RISC) (12) was used. The scale includes three dimensions: tenacity (13 items), self-improvement (8 items), and optimism (4 items), with a total of 25 items. Each item is scored on a 5-point Likert scale ranging from 0 to 4, resulting in a total score range of 0–100. Higher scores indicate better psychological resilience. In this study, the Cronbach’s α coefficient of the scale was 0.915.

#### 2.2.4 General Self-Efficacy Scale

The Chinese version of the General Self-Efficacy Scale (GSES), revised by Wang et al. (13), was adopted. The scale is one-dimensional and consists of ten items. Each item is scored on a 1–4 scale, with total scores ranging from 10–40. Higher scores indicate stronger self-efficacy. In this study, Cronbach’s α coefficient of the scale was 0.887.

#### 2.2.5 Stroke-Specific Quality of Life Scale

The revised Chinese version of the Stroke-Specific Quality of Life Scale (SS-QOL) (14) was used to assess QoL in patients with stroke. The scale includes 12 domains: energy, family roles, language, mobility, mood, self-care, social roles, thinking, upper extremity function, vision, work/productivity, and family responsibilities, comprising a total of 49 items. Each item is scored on a 1–5 scale. After formula conversion, total scores range from 0 to 100, with higher scores indicating better QoL. In this study, the Cronbach’s α coefficient of the scale was 0.934.

### 2.3 Data collection

Before data collection, five researchers received standardized training on the study’s purpose, questionnaire administration procedures, interpretation of scale items, communication skills, and quality control methods. Data collection was conducted only after all the researchers passed the training assessment.

A one-to-one survey approach was used. Researchers used standardized instructions to explain the study purpose, questionnaire completion methods, and confidentiality principles to the participants. After informed consent was obtained, the participants completed the questionnaires independently. For participants with physical limitations or writing difficulties, the researchers read each item aloud and recorded responses according to the participants’ verbal answers while avoiding leading or suggestive questions.

All questionnaires were distributed and collected on-site. After collection, the questionnaires were immediately checked for completeness. Questionnaires with a missing response rate greater than 10% or identical responses to all items were considered invalid and excluded from the analysis.

### 2.4 Statistical methods

The collected data were analyzed using SPSS version 22.0. The normality of continuous variables was assessed using the Shapiro–Wilk test. Normally distributed data are presented as mean ± standard deviation (x̄ ± s). Independent-samples *t* tests were used for comparisons between two groups, while one-way analysis of variance (ANOVA) with Bonferroni *post hoc* tests was used for comparisons among multiple groups. Categorical variables are presented as frequencies and percentages [n (%)].

Pearson’s correlation analysis was used to examine the relationships among the SSRS, CD-RISC, GSES, and SS-QOL scores. The PROCESS macro (Model 6) was applied to test the sequential mediation models. Age, education level, medical payment method, NIHSS score, time since stroke (months), and Charlson Comorbidity Index were entered as covariates to obtain the adjusted path coefficients and indirect effects. All reported results were adjusted for the covariates.

The significance of the indirect effects was evaluated using a bias-corrected bootstrap method with 5,000 resamples. A 95% confidence interval (CI) that did not include zero was considered indicative of a statistically significant mediating effect. Statistical significance was defined as a two-sided *p* value <0.05. Age, education level, medical payment method, NIHSS score, time since stroke (months), and Charlson Comorbidity Index were entered as covariates to obtain the adjusted path coefficients and indirect effects. All reported regression coefficients are unstandardized B coefficients. The significance of the indirect effects was evaluated using a bias-corrected bootstrap method with 5,000 resamples. A 95% confidence interval (CI) that did not include zero was considered indicative of a statistically significant mediating effect.

## 3 Results

### 3.1 Characteristics of the study participants

Among the 272 patients with severe stroke, 156 (57.35%) were male and 116 (42.65%) were female. The mean age was 62.35 ± 10.28 years (range: 38–79 years). Regarding marital status, 229 patients (84.19%) were married, while 43 (15.81%) were unmarried, divorced, or widowed. In terms of education level, 87 patients (31.99%) had a primary school education or below, 96 (35.29%) had a junior high school education, 58 (21.32%) had a senior high school or technical secondary school education, and 31 (11.40%) had a junior college education or above.

Regarding medical payment methods, 142 patients (52.21%) were covered by employee medical insurance, 121 (44.49%) by resident medical insurance, and 9 (3.30%) were self-funded patients. Ischemic stroke was diagnosed in 198 patients (72.79%), while 74 patients (27.21%) had a hemorrhagic stroke. The duration since stroke onset ranged from 1 to 6 months, with a mean duration of 3.25 ± 1.62. The NIHSS scores ranged from 10 to 24, with a mean score of 14.26 ± 3.18.

### 3.2 Scores of each scale

Among the 272 patients with severe stroke, the mean total SSRS score was 34.52 ± 7.18, including objective support (9.51 ± 2.28), subjective support (18.42 ± 4.19), and support utilization (6.59 ± 1.81) scores. The mean total CD-RISC score was 51.94 ± 11.34, including tenacity (28.57 ± 6.68), self-improvement (16.52 ± 4.12), and optimism (6.85 ± 2.10) scores. The mean total GSES score was 22.49 ± 5.43, and the mean total SS-QOL score was 51.65 ± 13.49. The detailed results are presented in **Table 1**.

**Table 1:** Score of each scale (x̄ ± s).

Item	Dimension	Score
SSRS	Total score	34.52 ± 7.18
MLHFQ	Objective Support	9.51 ± 2.28
	Subjective Support	18.42 ± 4.19
	Support Utilization	6.59 ± 1.81
CD-RISC	Total score	51.94 ± 11.34
	Toughness	28.57 ± 6.68
	Strength	16.52 ± 4.12
	Optimism	6.85 ± 2.10
GSES	Total score	22.49 ± 5.43
SS-QOL	Total score	51.65 ± 13.49

### 3.3 Comparison of SS-QOL scores among patients with severe stroke according to demographic and disease characteristics

Significant differences in SS-QOL scores were observed among patients with severe stroke across different age groups, education levels, medical payment methods, and NIHSS scores (*P* < 0.05). The detailed results are presented in **Table 2**.

**Table 2:** Comparison of SS-QOL scores in patients with severe stroke with different demographic and disease characteristics (x̄ ± s).

Characteristic	Grouping	Number of cases	SS-QOL	*t/F*	*P*	Effect size
Gender	Male	156	50.26 ± 12.78	1.600	0.111	0.196
	Female	116	52.87 ± 13.98			
Age	<60	98	54.62 ± 14.07	3.104	0.002	0.389
	≥60	174	49.34 ± 13.12			
Marital status	Married	229	52.04 ± 13.87	1.280	0.202	0.162
	Unmarried/divorced/widowed	43	49.87 ± 13.16			
Educational level	Junior high school or below	87	47.26 ± 11.38	4.715	0.010	0.034
	Senior high school or technical secondary school	154	51.23 ± 13.24^*^			
	College or above	31	54.73 ± 14.27^*^			
Medical payment method	Employee health insurance	142	53.26 ± 13.42	3.913	0.021	0.028
	Resident health insurance	121	50.15 ± 13.58^#^			
	Self-funded	9	42.36 ± 11.25^#^			
Stroke type	Ischemic	198	51.98 ± 13.56	0.897	0.551	0.082
	Hemorrhagic	74	50.89 ± 12.97			
NIHSS	10–14 points	152	56.38 ± 13.45	6.260	<0.001	0.763
	15–24 points	120	46.44 ± 12.41			

^*^ indicates comparison with primary school and below, *P* < 0.05; ^#^Indicates comparison with employee medical insurance, *P* < 0.05.

### 3.4 Correlation analysis between variables

**Figure 1:**
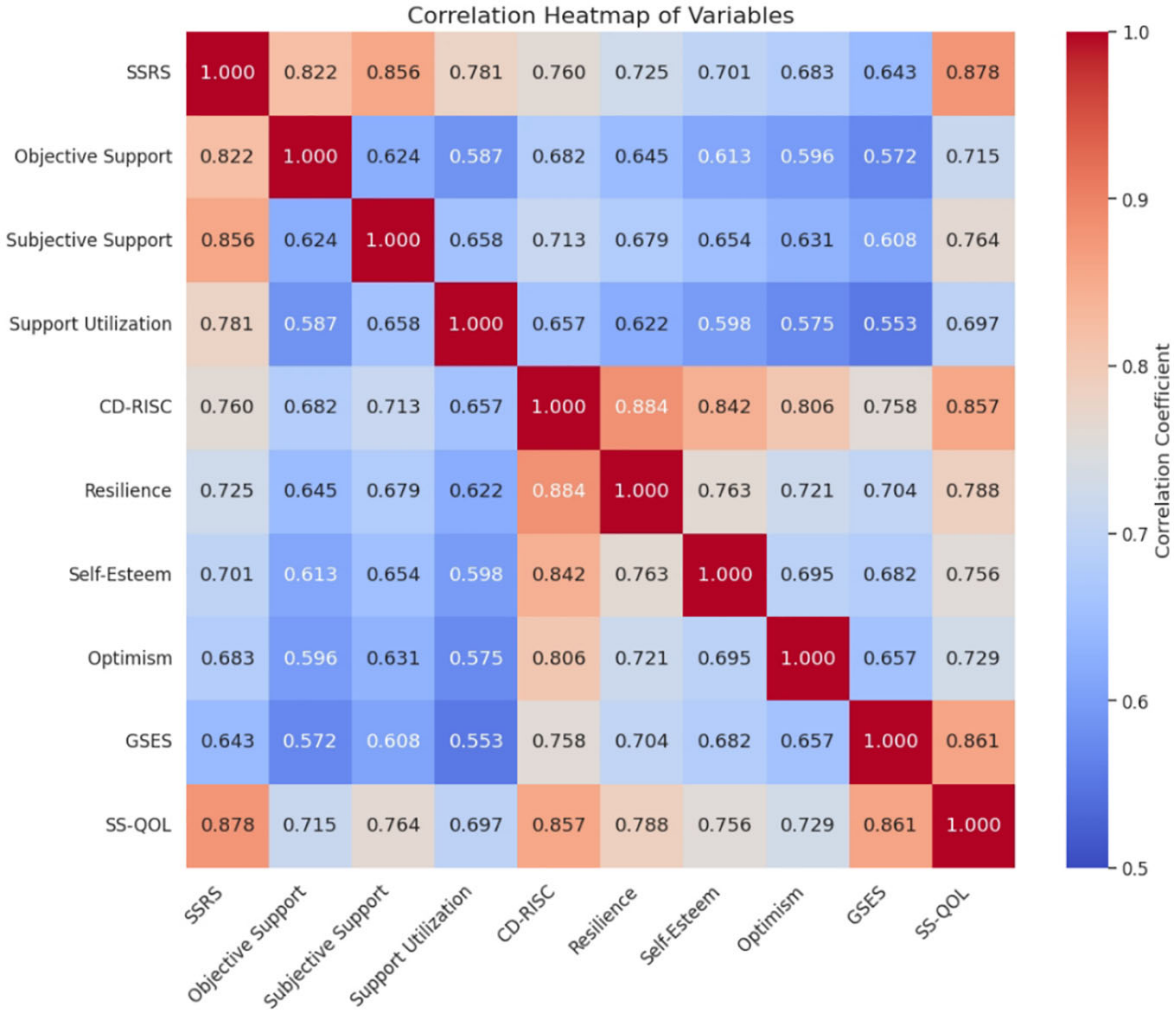
Correlation analysis between variables.

Pearson’s correlation analysis showed that the SSRS scores were significantly positively correlated with the CD-RISC, GSES, and SS-QOL scores (*r* = 0.760, 0.643, and 0.878, respectively; all *P* < 0.05). CD-RISC scores were also positively correlated with GSES and SS-QOL scores (*r* = 0.758 and 0.857, respectively; both *P* < 0.05). In addition, GSES scores were significantly positively correlated with the SS-QOL scores (*r* = 0.861, *P* < 0.05). The detailed results are presented in **Table 3**, **Figure 1**.

**Table 3:** Correlation analysis between variables.

Variable	1	2	3	4	5	6	7	8	9	10
1. SSRS	1	0.822	0.856	0.781	0.760	0.725	0.701	0.683	0.643	0.878
2. Objective Support	0.822	1	0.624	0.587	0.682	0.645	0.613	0.596	0.572	0.715
3. Subjective Support	0.856	0.624	1	0.658	0.713	0.679	0.654	0.631	0.608	0.764
4. Utilization of Support	0.781	0.587	0.658	1	0.657	0.622	0.598	0.575	0.553	0.697
5. CD-RISC	0.760	0.682	0.713	0.657	1	0.884	0.842	0.806	0.758	0.857
6. Tenacity	0.725	0.645	0.679	0.622	0.884	1	0.763	0.721	0.704	0.788
7. Strength	0.701	0.613	0.654	0.598	0.842	0.763	1	0.695	0.682	0.756
8. Optimism	0.683	0.596	0.631	0.575	0.806	0.721	0.695	1	0.657	0.729
9. GSES	0.643	0.572	0.608	0.553	0.758	0.704	0.682	0.657	1	0.861
10. SS-QOL	0.878	0.715	0.764	0.697	0.857	0.788	0.756	0.729	0.861	1

Pearson correlation analysis, all *P* < 0.05.

### 3.5 Mediating effects of resilience and self-efficacy in the relationship between social support and quality of life among patients with severe stroke

Using SSRS as the independent variable (*X*), SS-QOL as the dependent variable (*Y*), and CD-RISC and GSES as the mediating variables (*M1* and *M2*), a sequential mediation model was constructed. The model demonstrated that the SSRS positively predicted the SS-QOL, CD-RISC, and GSES scores, while the CD-RISC positively predicted the GSES score. In addition, both the CD-RISC and GSES showed significant positive predictive effects on the SS-QOL. The detailed results are presented in **Table 4**.

**Table 4:** Adjusted mediation model coefficients (PROCESS Model 6, controlling for age, education, payment method, NIHSS, time since stroke, and comorbidities).

Outcome variable	Predictive variable	Model coefficient			
		*β*	*SE*	*t*	*P*
SS-QOL	SSRS	0.882	0.047	18.767	<0.001
	CD-RISC	0.213	0.035	6.079	<0.001
	GSES	1.051	0.062	16.962	<0.001
CD-RISC	SSRS	1.200	0.063	19.205	<0.001
GSES	SSRS	0.120	0.046	2.617	0.009
	CD-RISC	0.306	0.029	10.560	<0.001

All coefficients are adjusted for age, education level, medical payment method, NIHSS score, time since stroke (months), and comorbidity count. The adjusted model showed consistent direct and indirect effect patterns compared to the unadjusted model.

### 3.6 Mediating effects of resilience and self-efficacy between social support and quality of life among patients with severe stroke

The direct effect of SSRS on SS-QOL was statistically significant (effect value = 0.882), accounting for 53.49% of the total effects. The mediating effects consisted of three pathways:

1. SSRS - CD-RISC - SS-QOL, with a mediating effect value of 0.255, accounting for 15.46% of the total effect.
2. SSRS - GSES - SS-QOL, with a mediating effect value of 0.126, accounting for 7.64% of the total effect.
3. The SSRS - CD-RISC - GSES - SS-QOL path had a mediating effect value of 0.385, accounting for 23.35% of the total effect.

**Figure 2:**
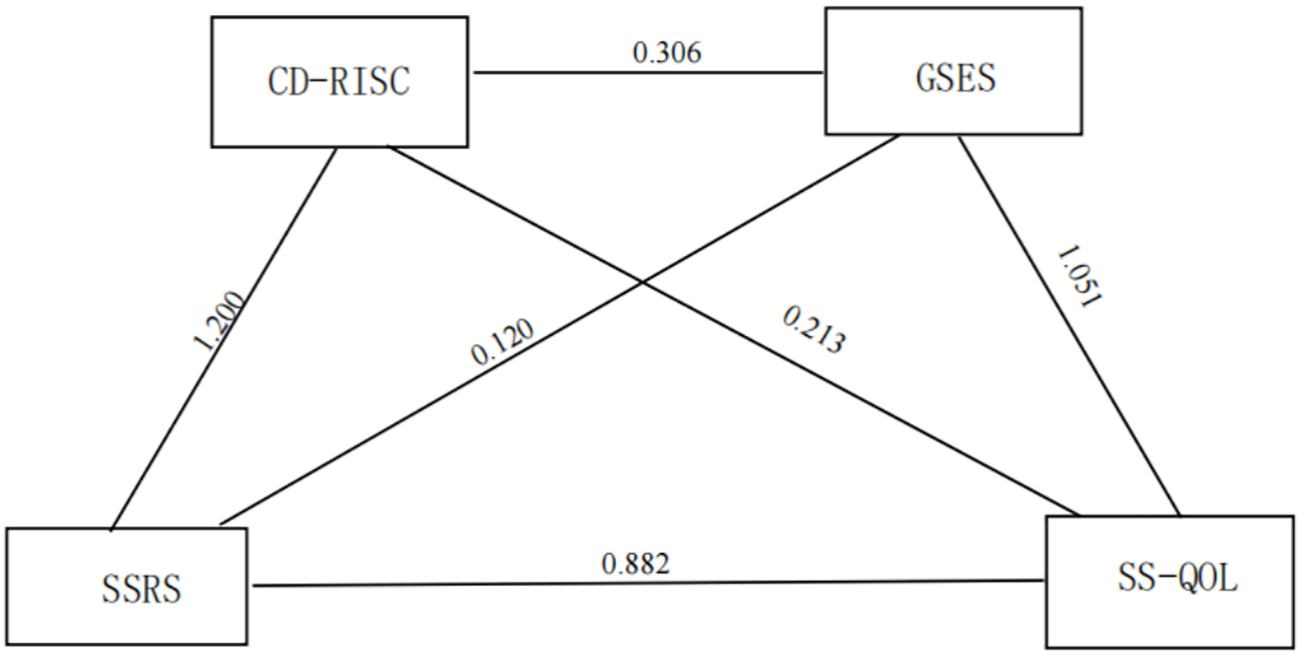
Mediating effect of resilience and self-efficacy between social support and quality of life in patients with severe stroke.

These findings suggest that resilience and self-efficacy exert significant chain-mediation effects on the relationship between social support and QoL among patients with severe stroke. The detailed results are presented in **Table 5**, **Figure 2**.

**Table 5:** The mediating effect of resilience and self-efficacy between social support and quality of life in patients with severe stroke.

Effect	Effect path	Effect value	*SE*	*95%CI*	Fully std.*β*	Effect ratio(%)
Total effect	SSRS-SS-QOL	1.649	0.055	1.541~1.756	0.638	100
Direct effect	SSRS-SS-QOL	0.882	0.047	0.790~0.975	0.341	53.49
Indirect effect	SSRS-CD-RISC-SS-QOL	0.255	0.035	0.192~0.329	0.099	15.46
	SSRS-GSES-SS-QOL	0.126	0.048	0.020~0.208	0.049	7.64
	SSRS-CD-RISC-GSES-SS-QOL	0.385	0.056	0.289~0.510	0.149	23.35

All effects are unstandardized regression coefficients (*β*). CI, bias-corrected bootstrap 95% confidence interval based on 5,000 resamples. All coefficients were adjusted for age, education level, medical payment method, NIHSS score, time since stroke (months), and Charlson Comorbidity Index.

## 4 Discussion

The results of this study showed that the mean SS-QOL score among patients with severe stroke was 51.65 ± 13.49, indicating a relatively low QoL, which is consistent with the findings of previous international studies (15). Patients with severe stroke often experience severe neurological deficits after onset, leading to impaired activities of daily living (ADL). Simultaneously, they are prone to negative emotions, such as anxiety and depression. In addition, the economic burden and changes in social roles caused by the disease may adversely affect patients’ physiological, psychological, and social well-being, thereby reducing their QoL. Importantly, given the cross-sectional design of this study, all reported associations should be interpreted as statistical associations, and no causal inferences can be drawn regarding the direction of relationships among social support, resilience, self-efficacy, and QoL. The following discussion should be read with this fundamental limitation in mind.

In this study, the mean social support score among patients with severe stroke was 34.52 ± 7.18, which was lower than that reported in the general community population (16), suggesting that these patients experience insufficient social support. After stroke onset, patients’ physical activity and social interactions are often restricted. The prolonged rehabilitation process may also impose substantial caregiving and financial burdens on families, thereby reducing the availability of family and social support for patients. In addition, resilience and self-efficacy scores were moderate to low, consistent with previous studies involving patients with severe stroke (17). Faced with major trauma and uncertainty regarding long-term rehabilitation, patients may experience feelings of helplessness and despair, leading to reduced confidence in their rehabilitation abilities and impaired psychological adaptation and recovery.

The results further showed significant differences in SS-QOL scores according to age, education level, medical payment method, and NIHSS score, suggesting that these demographic and disease-related factors are important determinants of QoL in patients with severe stroke. Younger and middle-aged patients generally have better baseline physical condition, stronger compensatory capacity of vital organs, and fewer chronic comorbidities. Therefore, they may have greater recovery potential and better rehabilitation outcomes after severe neurological injury. In contrast, older patients often experience slower recovery, more complications, and a higher risk of cognitive decline, which may reduce rehabilitation participation and self-management ability, ultimately impairing QoL (18). Patients with lower educational attainment may have limited disease-related knowledge and poorer rehabilitation awareness, which can contribute to inadequate rehabilitation adherence and negative illness perceptions, thereby affecting rehabilitation outcomes and QoL (19). Medical payment methods may also influence QoL through differences in economic burden and accessibility of healthcare resources. Patients covered by employee medical insurance generally have higher reimbursement rates and broader coverage, which may facilitate access to standardized rehabilitation services and supportive care, thereby improving functional recovery and QoL. Higher NIHSS scores indicate more severe neurological impairment, including hemiplegia, speech disorders, swallowing dysfunction, and cognitive impairment, which can markedly reduce self-care ability, physical activity, and social participation. Severe neurological deficits may also increase the risk of complications, such as pulmonary infection, pressure ulcers, deep venous thrombosis, and refractory epilepsy, further reducing QoL (20).

Pearson’s correlation analysis demonstrated significant positive correlations among the SSRS, CD-RISC, GSES, and SS-QOL scores (all *P* < 0.05). SSRS was positively correlated with SS-QOL, indicating that patients who received higher levels of social support tended to report better QoL. Adequate social support may provide emotional comfort, material assistance, and rehabilitation care, thereby reducing psychological stress and encouraging active participation in the rehabilitation process. SSRS was also positively correlated with the CD-RISC and GSES, suggesting that greater social support may enhance resilience and self-efficacy. Support and encouragement from family members, friends, and healthcare professionals may help patients better accept their disease, reduce negative perceptions, strengthen psychological adaptation, and increase confidence in rehabilitation (21). Furthermore, CD-RISC and GSES scores were positively correlated with SS-QOL scores, indicating that patients with higher resilience and self-efficacy tended to have better QoL. Patients with greater resilience are more likely to maintain a positive attitude and adopt adaptive coping strategies during rehabilitation, whereas patients with stronger self-efficacy are more likely to actively participate in rehabilitation training and disease self-management, thereby improving their neurological function and QoL (22).

The mediation analysis showed that the direct effect of the SSRS on the SS-QOL remained significant after controlling for the mediating effects of the CD-RISC and GSES, accounting for 53.49% of the total effect. This finding suggests that social support is an important factor associated with improved QoL in patients with severe strokes. Objective support may reduce the financial burden of medical care and improve access to rehabilitation resources and caregiving services, thereby directly improving patients’ physical function and daily living conditions. Subjective emotional support may alleviate loneliness and helplessness, improve psychological well-being, and enhance QoL. In addition, social participation and interpersonal support may help patients maintain social roles and reduce feelings of isolation, thereby improving their social functioning and QoL (23). These findings highlight the importance of establishing comprehensive social support systems for patients with severe stroke through family education, healthcare communication, community rehabilitation, and social assistance services.

CD-RISC showed a significant mediating effect between social support and QoL, accounting for 15.46% of the total effect. This finding suggests that social support may be associated with improved QoL partly through enhanced resilience. As an important external protective factor, social support may buffer disease-related stress by strengthening patients’ internal psychological resources. Adequate social support can provide emotional security and rehabilitation encouragement, helping patients establish more positive cognitive appraisals, reduce catastrophic thinking, and strengthen optimism and psychological adaptability (24). Patients with higher resilience are better able to cope with physical and psychological changes caused by severe stroke and are more likely to actively engage in rehabilitation activities, which may contribute to better QoL.

The GSES also demonstrated a significant mediating effect between social support and QoL, accounting for 7.64% of the total effect. According to social cognitive theory, self-efficacy is influenced by verbal encouragement, observational learning, and successful experiences, all of which may be facilitated by social support. Encouragement and affirmation from family members and healthcare professionals may help patients recognize their rehabilitation potential, gain rehabilitation-related knowledge and skills, and accumulate successful rehabilitation experiences, thereby enhancing their self-efficacy. Higher self-efficacy may further promote positive rehabilitation behaviors, improve rehabilitation adherence and disease self-management, reduce negative emotions, and ultimately improve QoL (25).

Importantly, this study identified a significant chain mediating effect of resilience and self-efficacy between social support and QoL, accounting for 23.35% of the total effects. The findings suggest the following pathway: social support - resilience - self-efficacy - QoL. Social support, as an external environmental resource, may first strengthen resilience, thereby enhancing patients’ psychological adaptability and recovery. Higher resilience may subsequently improve self-efficacy by helping patients maintain a positive rehabilitation attitude, actively overcome rehabilitation difficulties, and accumulate successful experiences during recovery (26). Enhanced self-efficacy may encourage active participation in rehabilitation and improve self-management ability, ultimately contributing to a better QoL.

These findings extend previous research by clarifying the sequential and chain-mediating roles of resilience and self-efficacy in the relationship between social support and QoL. Compared with single mediation models, the chain mediation model more comprehensively explains how external social support may be transformed into internal psychological motivation, which promotes better health outcomes. These findings suggest that psychological interventions for patients with severe stroke could potentially benefit from a sequential strategy that first focuses on enhancing resilience and then strengthening self-efficacy. However, this hypothesis requires testing in longitudinal and intervention-based studies before any clinical recommendations can be made. If future research confirms the temporal precedence of these pathways, interventions that strengthen social support systems and target resilience and self-efficacy sequentially may be associated with better rehabilitation outcomes.

Several limitations should be considered when interpreting the findings of this study. First, the cross-sectional design precludes any causal inferences. Although our hypothesized sequential model (social support - resilience - self-efficacy - QoL) was grounded in social cognitive theory and prior empirical research, we cannot empirically establish the temporal ordering of these variables. It is entirely plausible that: (a) the relationship between resilience and self-efficacy is bidirectional, with self-efficacy enhancing resilience through repeated successful coping experiences, and resilience enhancing self-efficacy through maintaining a positive orientation toward recovery; (b) self-efficacy may serve as an antecedent rather than a consequence of resilience in some patients; or (c) the observed associations may be explained by reverse causation—for example, patients with better QoL may be more likely to perceive higher social support and report greater resilience and self-efficacy. Consequently, our findings should be interpreted as supporting a statistical mediation model—that is, resilience and self-efficacy are jointly associated with the social support-QoL relationship—rather than a causal mediation process. The chain sequence we propose is one plausible explanation among several alternatives. We emphasize that the present results at most support a mediating role for resilience and self-efficacy in the relationship between social support and QoL, but do not establish the directionality of these associations. Second, although several important demographic and disease-related covariates were adjusted for, residual confounding from unmeasured variables such as personality traits, cognitive status, rehabilitation intensity, and social desirability bias cannot be excluded. Third, the study was conducted in a single tertiary hospital in Shandong Province, China, which may limit the generalizability of our findings. The cultural characteristics of Chinese society, including strong family centered support systems and collectivist values, may influence the expression of social support, resilience, and self-efficacy. Therefore, caution is warranted when generalizing these findings to other cultures. Future longitudinal, multicenter, and cross-cultural studies are needed to further validate the identified mediation pathways and to improve the generalizability of the findings.

In conclusion, social support was significantly associated with QoL, both directly and indirectly, through resilience, self-efficacy, and their sequential pathways. These findings highlight resilience and self-efficacy as promising psychological targets that may be associated with better QoL in patients with severe stroke. In clinical practice, rehabilitation programs that seek to strengthen social support systems while incorporating psychological components to enhance resilience and self-efficacy may be beneficial. However, given the cross-sectional nature of this study, we emphasize that these are associational findings, and any clinical recommendations must be further validated through longitudinal or intervention-based research designs before causal claims can be made.

## Data Availability

The original contributions presented in the study are included in the article/supplementary material. Further inquiries can be directed to the corresponding author.
